# The Lifespan of *D. melanogaster* Depends on the Function of the *Gagr* Gene, a Domesticated *gag* Gene of Drosophila LTR Retrotransposons

**DOI:** 10.3390/insects15010068

**Published:** 2024-01-17

**Authors:** Yevgenia Balakireva, Maria Nikitina, Pavel Makhnovskii, Inna Kukushkina, Ilya Kuzmin, Alexander Kim, Lidia Nefedova

**Affiliations:** 1 Department of Genetics, Lomonosov Moscow State University, Leninskie Gory 1, 119991 Moscow, Russia; balakireva.evgesha@mail.ru (Y.B.); masha-nn23@yandex.ru (M.N.); vladimirova-bph@yandex.ru (I.K.); kuzmin.ilya@gmail.com (I.K.); aikim57@mail.ru (A.K.); 2 Institute of Biomedical Problems, Russian Academy of Sciences, 123007 Moscow, Russia; maxpauel@gmail.com; 3 Faculty of Biology, Shenzhen MSU-BIT University, Longgang District, Shenzhen 518172, China

**Keywords:** *Drosophila*, signaling pathway, domesticated retroviral *gag* gene, immunity, ammonium persulphate

## Abstract

**Simple Summary:**

Transposable elements serve as a potent genetic resource for the host genome, playing a key role in the formation of diverse regulatory sequences and new genes. The evolutionary process of adaptation of transposable element sequences by a host for its own benefit is termed ‘molecular domestication’. Among genetic model organisms, *Drosophila melanogaster* is extensively used for studying LTR retrotransposons, a class I of transposable elements present in diverse groups within its genome. Nonetheless, the molecular domestication of LTR retrotransposons in *D. melanogaster* remains underexplored. Our study focuses on the role of the domesticated LTR retrotransposon capsid *gag* gene, *Gagr*, in the *D. melanogaster* genome. We conducted a comparative analysis of flies with a *Gagr* gene knockdown in all tissues against control flies through physiological testing and RNA-sequencing experiments. The flies with the *Gagr* gene knockdown demonstrated a reduced lifespan compared to control flies. At the same time, flies with the *Gagr* gene knockdown exhibited altered transcription patterns in categories of genes related to developmental control, morphogenesis, and central nervous system functionality. Our findings highlight the crucial role of the *Gagr* gene in maintaining immune response and homeostasis.

**Abstract:**

(1) Background: The *Gagr* gene in *Drosophila melanogaster*’s genome originated from the molecular domestication of retrotransposons and retroviruses’ *gag* gene. In all *Drosophila* species, the Gagr protein homologs exhibit a conserved structure, indicative of a vital role. Previous studies have suggested a potential link between the *Gagr* gene function and stress responses. (2) Methods: We compared flies with *Gagr* gene knockdown in all tissues to control flies in physiological tests and RNA-sequencing experiments. (3) Results: Flies with the *Gagr* gene knockdown exhibited shorter lifespans compared to control flies. Transcriptome analysis revealed that *Gagr* knockdown flies showed elevated transcription levels of immune response genes. We used ammonium persulfate, a potent stress inducer, to elicit a stress response. In control flies, ammonium persulfate activated the Toll, JAK/STAT, and JNK/MAPK signaling pathways. In contrast, flies with the *Gagr* gene knockdown displayed reduced expression of stress response genes. Gene ontology enrichment analysis identified categories of genes upregulated under ammonium persulfate stress in control flies but not in *Gagr* knockdown flies. These genes are involved in developmental control, morphogenesis, and central nervous system function. (4) Conclusion: Our findings indicate the significance of the *Gagr* gene in maintaining immune response and homeostasis.

## 1. Introduction

Molecular domestication of retroelements, including retrotransposons and retroviruses, is a significant factor in forming new genes in eukaryotic genomes. All three genes of retroelements with long terminal repeats (LTRs)—*gag*, *pol*, and *env*—domesticated homologs with functions beneficial to the host organism have been identified. The capsid gene, *gag*, exhibits the greatest diversity in such genes. In mammals, *gag* domestication is exemplified by gene families like *PNMA* (ParaNeoplastic Ma Antigens), MART (Mammalian RetroTransposons), and *SIRH* (Sushi-Ichi Retrotransposon Homologues) [1]. The *PNMA* family includes genes that regulate apoptosis [2], while many *MART*/*SIRH* family genes are expressed in the placenta, playing vital roles in its early formation and development [3]. Another notable example of *gag* domestication is the SCAN domain, which is prevalent among *Tetrapoda* transcription factors and influences various biological processes such as embryonic development, hematopoiesis, and metabolism [4]. Additionally, several domesticated *gag* genes contribute to retrovirus protection, as observed in mice with genes like *Fv1*, *Fv4*, *Rmcf1*, and *Rmcf2* [5]. Recent characterizations of domesticated retroelement sequences include the *LINE* retroelement upstream of the *Pparg* gene, essential for adipogenesis [6], and the *PRLH1* transcript from the endogenous retrovirus ERV-9, involved in repairing double-strand breaks [7].

The *Drosophila melanogaster Gagr* gene serves as an invertebrate example of the molecular domestication of the *gag* gene from retrotransposons/retroviruses [8]. In all sequenced *Drosophila* genomes, *Gagr* genes possess a highly conserved structure, reflecting long-term domestication under stabilizing selection [8]. The function of the *Gagr* gene remains unknown.

Studies suggest a potential link between the *Gagr* gene and immune responses or stress-related processes [9]. *Gagr* gene research indicates its involvement in several crucial processes associated with stress reactions. For example, bacterial lipopolysaccharides induce *Gagr* expression in S2 cells, dependent on MAPK/JNK stress signaling pathway regulators Tak1, hep, and bsk [10]. Additionally, intrabdominal injection of viruses like DCV, FHV, and SINV significantly increases *Gagr* expression [11]. We previously identified a binding motif for the kayak transcription factor, a component of the JNK signaling pathway, and two motifs for the Stat92E transcription factor, part of the JAK/STAT pathway, in the *Gagr* gene promoter [9].

Protein-protein interactions of Gagr were established in *D. melanogaster* S2R+ cells [12]. The Gagr protein interacts with five proteins (CG3687, CG6013, 14-3-3e, Pdi, and eIF3j); most of them have stress-related functions [8]. 14-3-3e regulates MAPK and other stress pathways [13]; Pdi is crucial in endoplasmic reticulum stress and the unfolded protein response [14]; eIF3j is necessary for IRES-dependent translation during cell stress [15]. The *CG3687* gene, less studied in *D. melanogaster*, is associated with a flightless phenotype when knocked down [16]. The function of the CG6013 protein, homologous to the human CCDC124 protein, remains unknown, but in *Saccharomyces pombe*, the orthologous *Oxs1* gene product is a cofactor in the Pap1/Oxs1 signaling pathway [17]. Therefore, investigating the *Gagr* gene’s role in cell stress, given its activation under stress and protein interactions, is crucial.

To study the *Gagr* gene’s function, we employed the reduction of *Gagr* transcription through RNA interference-induced knockdown. The flies with *Gagr* gene knockdown in all tissues were tested for their lifespan, motility, and transcriptomic responses to ammonium persulfate (APS), a cellular homeostasis disruptor.

## 2. Materials and Methods

### 2.1. Drosophila Melanogaster Strains and Cultivation Conditions

The following strains of *D. melanogaster* were used: w^1118^; tub-GAL4, driver strain from Bloomington Drosophila Stock Center (*y^1^*, *w*^1118^; P{*w*^+mC^ = tubP-GAL4}LL7 P{ry^+t7.2^ = neoFRT}82B/TM6B, *Tb*^1^); P{KK109908}VIE-260B, KK RNAi strain from the Vienna Drosophila Resource Center, carrying a transgenic construct for expression of dsRNA for the *Gagr* gene RNA interference under UAS region control. Fly stocks were maintained in a standard nutrient agar medium at 25 °C. To induce interference, females of the UAS-Gagr RNAi strain were crossed with males of the tub-GAL4 driver strain. Thus, analyzed hybrids with knockdown of the *Gagr* gene, which we called Gagr^RNAi^, were obtained. Females of the w^1118^ strain were crossed with tub-GAL4 males as a control.

### 2.2. Physiological Tests

Lifespan was measured at 27 °C on the standard medium and on the medium supplemented with 0.1 M APS. To analyze lifespan flies at the age of 1 day were selected, separated by sex, and put into separate test tubes of 20–30 flies. The number of individuals in the test tube was checked every 2–3 days (or hours—for APS medium); the food was replaced every 5 days. To measure the motility of the imago, the climbing test was used [18] with modifications: 30 adults were placed in an empty long tube 17 cm long. Flies were dropped to the bottom of the test tube by mechanical tapping. Next, the time it took for each fly to achieve its maximum vertical climb rate was measured. Two independent repeats were performed.

### 2.3. RNA Extraction and RT-PCR

Total RNA was isolated from the Gagr^RNAi^ and control females and males (5–7 days old) after standard cultivation or after 24-h exposure to 0.1 M APS. RNA was isolated from pools (five females or seven males) in 5–7 biological replicates using the ExtractRNA reagent (Evrogen, Moscow, Russia), according to the manufacturer’s protocol; then, it was treated with DNase I (Thermo Fisher Scientific, Waltham, MA, USA). Reverse transcription was carried out using an MMLV-RT kit (Evrogen), according to the manufacturer’s protocol, with random primers (Evrogen). For quantitative PCR with the obtained cDNA, a Taq polymerase-based reaction mixture with SYBR Green I (Evrogen) was used in accordance with the manufacturer’s protocol. The reaction was performed using a Mini Opticon Real-Time PCR System (Bio-Rad Laboratories, Hercules, CA, USA). The relative expression of the genes *Gagr*, *TotA*, *TotC*, *AttB*, *CecA2*, *Socs16D*, *Spn88Eb*, *CG33346*, *Nazo*, *Ppo1*, *Spn28Dc*, *CG1304*, *CG10232*, *Ser6*, *CG10051*, normalized to the expression of two reference genes, *Tub84D* and *EloB*, was analyzed. Amplification was performed with primers shown in Table S3. A histogram was constructed in the GraphPad Prism 9 program (https://www.graphpad.com/) to present the expression results. Statistical significance was assessed using the nonparametric Mann–Whitney test in GraphPad Prism 9.

### 2.4. RNA-Sequencing and Data Processing

RNA was isolated from pools (five females or seven males) in 5–7 biological replicates using the ExtractRNA reagent (Evrogen), according to the manufacturer’s protocol, and then it was treated with DNase I (Thermo Fisher Scientific). RNA concentration and integrity were evaluated by a fluorimetric assay with Qubit 4 (Thermo Fisher Scientific) and capillary electrophoresis on Tape Station (Agilent Technologies, Santa Clara, CA, USA), respectively. All samples were prepared in one experiment (3 repeats for each sample). Strand-specific libraries were prepared by the NEB Next Ultra II Directional RNA Library Preparation kit (NEB, Ipswich, MA, USA) and sequenced (100 nucleotides, single end) with a median depth of 25 million reads per sample by NovaSeq 6000 (Illumina, San Diego, CA, USA). Low-quality reads, and adapter sequences were deleted (Timmomatic tool, v0.36), then the reads were aligned to the BDGP6 primary genome assembly. Uniquely aligned reads were counted for known exons of each gene using the R package (R environment). For the reference genes *ppl*, *Tbp*, *Gapdh1*, *tub*, *RPL40*, and *SdhA*, the expression deviation for each gene in the sample was assessed and normalized to the expression value of the corresponding gene in the control samples without exposure to APS. Deviations for different genes averaged +/−0.3. Differential expression analysis was performed by the DESeq2 package (version 1.41.0) (https://bioconductor.org/packages/release/bioc/html/DESeq2.html (accessed on 30 April 2023)). The heatmap of the differentially expressed genes is done using the heatmap package (https://www.bioinformatics.com.cn/plot_basic_cluster_heatmap_plot_024_en (accessed on 3 October 2023); [19]). Differentially expressed genes (DEGs) were protein-coding genes with |Log2 Fold Change| ≥ 0.6, Padj < 0.05. Metascape analysis tools were used to identify functional enrichment categories of DEGs (http://metascape.org (accessed on 3 October 2023); [20]). The Gene Ontology Resource (https://geneontology.org/ (accessed on 3 October 2023)) was used to search for molecular function enrichment. To determine tissues associated with the transcriptome response, we used data from the FlyAtlas 2 project [21]. We assessed correlations of tissue-specific expression values for DEGs and built a gene co-expression network for correlations of 0.8 or more using Cytoscape analysis based on the graph-oriented clustering method MCODE (https://cytoscape.org/ (accessed on 3 October 2023)). 

## 3. Results

### 3.1. Physiological Tests of the Gagr Gene Knockdown Flies 

#### 3.1.1. Knockdown of the *Gagr* Gene Does Not Affect Embryonic and Larval Viability of Flies

To initiate the *Gagr* gene RNA interference, we crossed females from the VDRC KK strain P{KK109908}VIE-260B, which carry a transgenic construct for dsRNA expression of the *Gagr* gene fragment under UAS region control, with males from the tub-GAL4 driver strain. For controls, females of the w^1118^ strain were crossed with tub-GAL4 males. The tub-GAL4 driver strain is heterozygous for the dominant *Tubby* mutation, located on the third chromosome and characterized by a short body phenotype and extra macrochetes on both sides of the head. Opposite the *Tubby* allele, on the homological chromosome, the *GAL4* gene is located, which is essential for RNA interference induction. The genetic construct enabling *Gagr* gene knockdown is located on the second chromosome of the responder strain. Crossbreeding of the driver and responder strains should result in offspring with a 1:1 ratio of long-bodied knockdown and short-bodied flies as a byproduct. To validate the 1:1 hypothesis, we employed the chi-square (χ^2^) method (Table 1). For the control hybrids, χ^2^ = 3.14, α = 0.076. Similarly, in the *Gagr* knockdown hybrids, χ^2^ = 3.52, α = 0.061. Therefore, the knockdown of the *Gagr* gene does not influence the embryonic and larval viability of the flies.

#### 3.1.2. Knockdown of the *Gagr* Gene Affects the Lifespan of Flies under Standard and Stress Conditions

**Figure 1:**
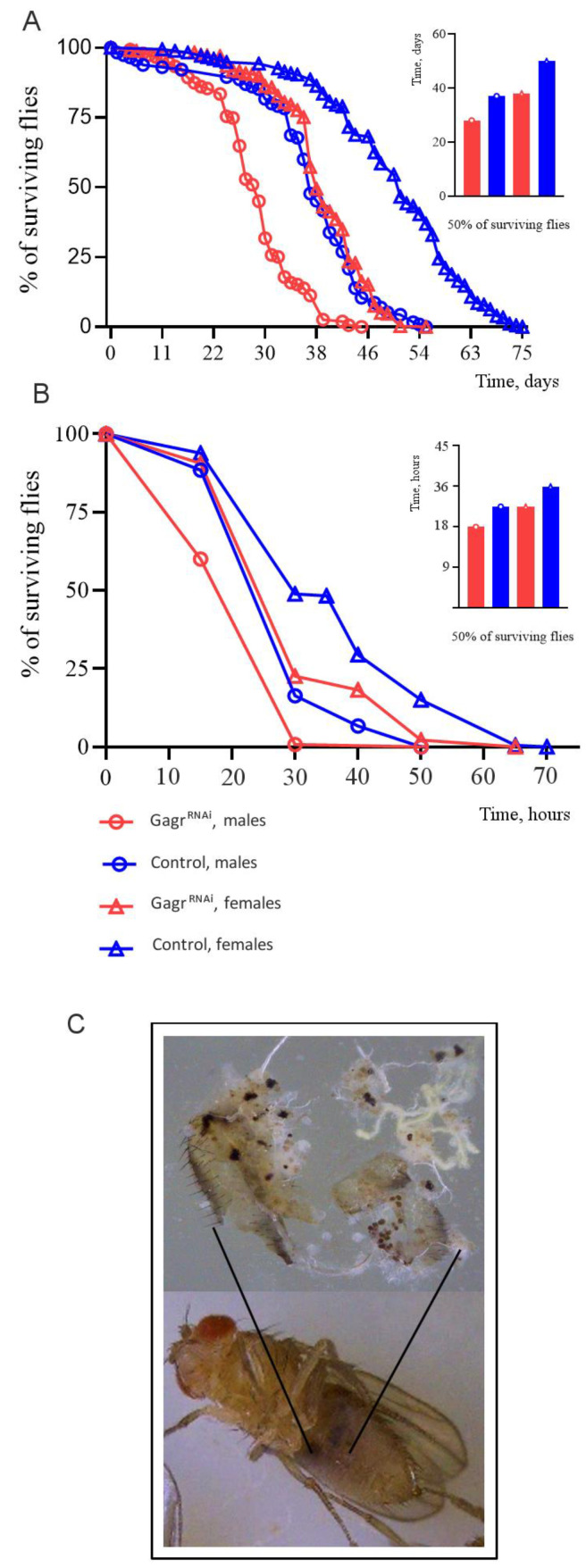
Physiological characteristics of the Gagr^RNAi^ flies. (**A**) The lifespan of the control and Gagr^RNAi^ flies under standard conditions at 27 °C. N = 115 for males, and N = 176 for females. (**B**) The survival rate of the control and Gagr^RNAi^ flies on a medium containing 0.1 M APS. N = 45 for males and females. (**C**) Melanin granules are found in the fat body of females.

We investigated the lifespan of *Gagr* knockdown flies (hereafter referred to as Gagr^RNAi^) compared to control flies (Figure 1A). The maximum lifespan observed for Gagr^RNAi^ males was 45 days, while control males reached up to 55 days. Similarly, Gagr^RNAi^ females had a maximum lifespan of 55 days compared to 75 days for control females. Consequently, Gagr^RNAi^ flies exhibited a reduced maximum lifespan compared to the control group.

Subsequently, we assessed the survival rate of flies on a medium containing 0.1 M APS. Under these conditions, the maximum lifespan for Gagr^RNAi^ males was 30 h, and for females, it was 50 h (Figure 1B). In comparison, control males and females had maximum lifespans of approximately 50 h and 64 h, respectively. Therefore, under APS-induced stress, Gagr^RNAi^ flies (both males and females) demonstrated a decreased survival rate compared to controls. Notably, the females showed greater resilience to APS stress than males.

#### 3.1.3. Knockdown of the *Gagr* Gene Does Not Lead to Changes in Adult Motility

Flybase data indicated that the knockdown of the *CG3687* gene, which encodes a protein interacting with the Gagr protein, results in a flightless phenotype (refer to Flybase report FBgn0034097). We hypothesized that the knockdown of the *Gagr* gene might impact the function of the CG3687 protein, thereby affecting the motility of flies. However, our experiments revealed no significant difference in vertical ascent time between the *Gagr* gene knockdown flies and control flies. All tested individuals were able to cover a distance of 17 cm in approximately 10 ± 1 s. Therefore, our findings suggest that the knockdown of the *Gagr* gene does not affect fly motility.

#### 3.1.4. Knockdown of the *Gagr* Gene in Females Promotes the Occurrence of Melanin Capsules in the Fat Body

Although no morphological changes were evident in the overall appearance due to the *Gagr* gene knockdown, we observed that only the females, not the males, developed black spots visible through the abdominal cuticle. Upon dissection, these spots were identified as multiple black granules, or melanin capsules, within the fat body (Figure 1C). Interestingly, similar phenomena were not observed in males with the *Gagr* gene knockdown.

### 3.2. Transcriptomic Analysis of the Gagr Gene Knockdown Flies

#### 3.2.1. Differentially Expressed Genes in the *Gagr* Knockdown Flies during Normal and Stress Conditions

**Figure 2:**
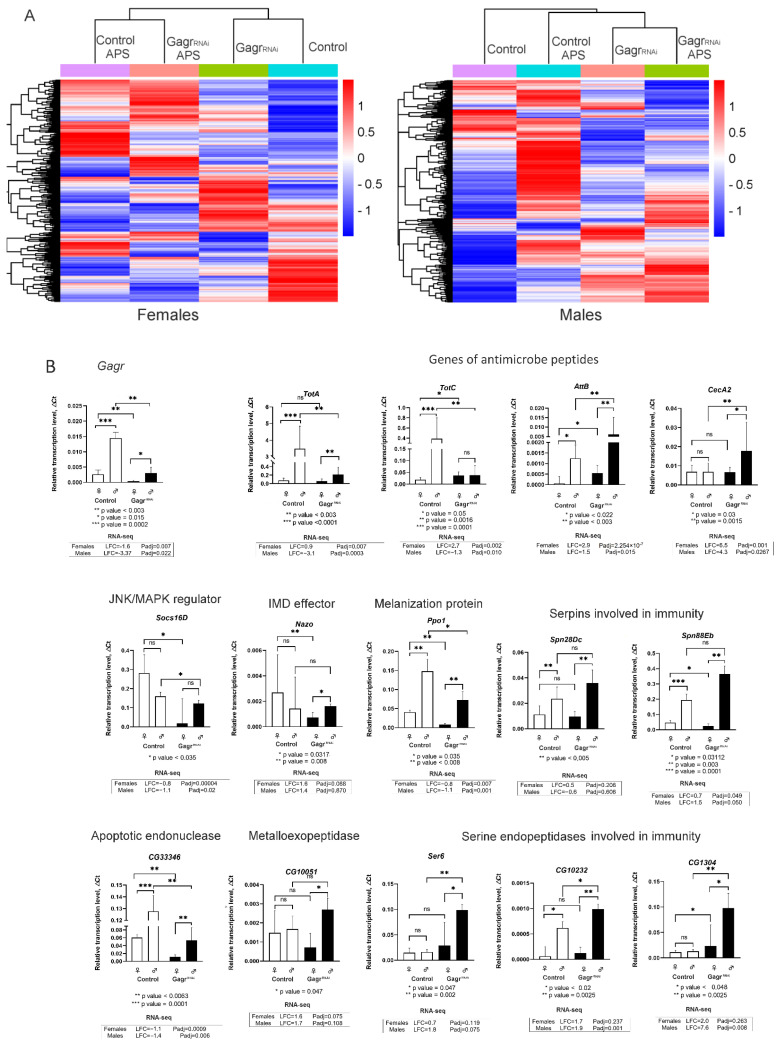
Differently expressed genes in the Gagr^RNAi^ and control flies. (**A**) Heatmap of transcriptomes of the control and Gagr^RNAi^ flies during standard and APS stress conditions. (**B**) RT-PCR analysis of transcription level of *Gagr* and 14 immune response genes with differential expression according to RNA-seq in Gagr^RNAi^ and control flies. Above diagrams, ns—not significant. Under diagrams, LFC values for RNA-seq data are shown.

We conducted a comparative analysis of the transcriptomes of control and Gagr^RNAi^ adult flies under both normal and APS stress conditions (Table S1). For eight datasets (control females, control males, Gagr^RNAi^ females, Gagr^RNAi^ males, each under both stress and normal conditions), we constructed a heatmap of average gene expression, as depicted in Figure 2A. This heatmap clearly demonstrates that the expression patterns of identified DEGs can effectively distinguish between the four types of samples for both females and males. In females, the transcriptomes of control and Gagr^RNAi^ flies are similar under both normal and APS stress conditions (Figure 2A). In males, however, the transcriptomes are clustered not by the presence or absence of the *Gagr* gene but rather by the environmental conditions, suggesting that *Gagr* knockdown influences the male transcriptomic response to APS stress.

Given the absence of a universally accepted fold change threshold for defining DEGs due to the variation in transcription rates among genes, we opted to set an initial threshold at 1.5. Under normal conditions, among approximately 13.5 thousand genes, 509 exhibited a change in transcription level of 1.5-fold or more (|Log2FoldChange(LFC)| > 0.6, Padj < 0.05) in females, and 346 in males with *Gagr* gene knockdown. Specifically, in Gagr^RNAi^ females, 297 genes showed increased transcription levels compared to control females, while in males, 191 genes were upregulated. Conversely, the transcription of 263 genes was downregulated in Gagr^RNAi^ females relative to controls and 153 genes in Gagr^RNAi^ males. Notably, 34 genes showed increased transcription in both sexes, including genes for antimicrobial peptides Attacins and Drosomicins, regulated by the Imd and Toll signaling pathways. Similarly, the transcription of 28 genes was reduced in both sexes, comprising genes such as *Lsp1beta*, *MtnE*, *Cyp4d1*, *Yp1*, and *Mal*-*B1*, which are involved in metabolic processes. These changes in gene expression may account for the observed alterations in lifespan in both females and males.

Upon APS exposure, we noted significant transcriptional changes in a large number of genes in both sexes (Table S1). In control females, 418 genes showed increased expression, while 795 exhibited decreased expression (|LFC| > 0.6, Padj < 0.05). In Gagr^RNAi^ females, 411 genes increased, and 458 genes decreased their expression. Among control males, 171 genes increased, and 44 genes decreased their expression, while in Gagr^RNAi^ males, 67 genes showed increased expression, and 42 genes showed decreased expression.

To establish the threshold for assessing DEGs, we conducted RT-PCR verification of RNA-seq data for the Gagr gene and 14 DEGs related to immune response (Figure 2B). The PCR analysis encompassed DEGs in both control and Gagr^RNAi^ flies, including the *Gagr* gene itself; antimicrobial peptide genes *TotA*, *TotC*, *AttB*, and *CecA2*, highly expressed in the fat body; the *Socs16D* gene, a repressor of the JNK/MAPK pathway; the *Nazo* gene, an antiviral effector of the Imd pathway; serine endopeptidase inhibitor genes *Spn88Eb* and *Spn28Dc*, involved in regeneration and melanization inhibition; serine endopeptidase genes *Ser6*, *CG1304*, and *CG10232*; the metalloexopeptidase gene *CG10051*; the apoptotic endopeptidase gene *G33346*; and *Ppo1*, a major gene for prophenoloxidase involved in melanization.

These genes were selected based on their differential expression levels in males and females, with LFC values greater than 0.6 and varied *p*-values, including some with |LFC| > 0.6 and *p*-value > 0.05. Since the cDNA library and RNA-seq data were derived from a single experiment, we performed verification for four groups: control and Gagr^RNAi^ males and females, analyzing 60 samples in total. PCR for each sample was conducted in 5–7 biological replicates.

In most instances, our PCR results corroborated the RNA-seq data, demonstrating good agreement between RNA-seq *p*-values and the reliability of PCR findings. Notably, the accuracy of expression level determination was influenced by gene expression levels (high or low) and the variability of expression. Genes like *CecA2* (males), *Nazo*, and *CG10051* showed differential expression in RNA-seq but had non-significant *p*-values. We encountered only two discrepancies between PCR and RNA-seq results: in females, PCR analysis did not confirm RNA-seq data for the *TotA* and *CecA2* genes. These inconsistencies might be attributed to experimental errors due to limited gene coverage or slight variances in gene expression between younger and older females used in the study (ages 5–7 days).

**Figure 3:**
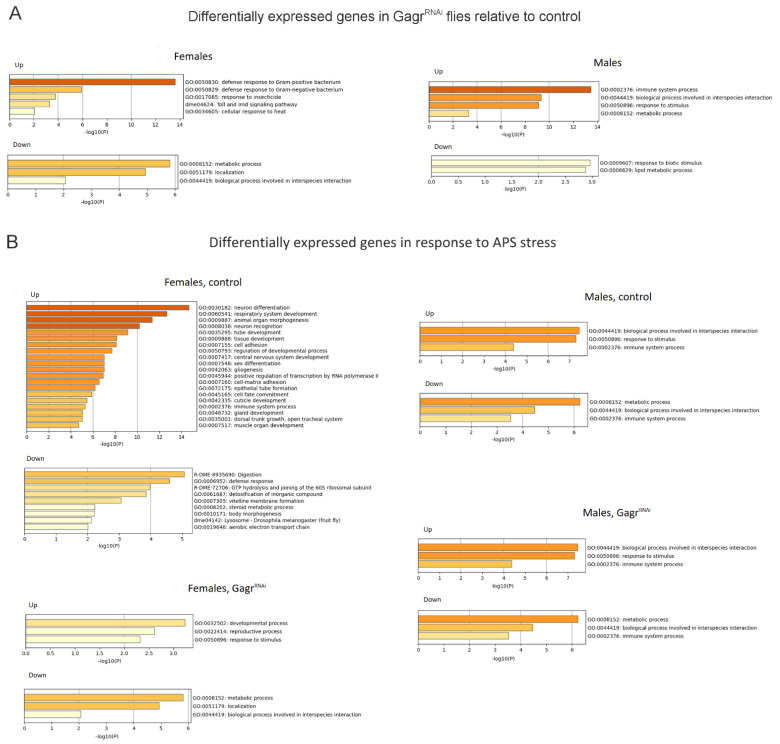
Enrichment of DEGs in the Gagr^RNAi^ and control females and males by functional categories of genes (LFC| > 1, Padj < 0.05). (**A**) DEGs in the Gagr^RNAi^ flies relative to control in normal conditions. (**B**) DEGs in the Gagr^RNAi^ flies, and control flies in response to APS exposure relative to normal conditions.

For enrichment analysis, we set the thresholds at |LFC| > 1 and Padj < 0.05. Raising the threshold did not alter the enrichment; however, it eliminated categories that exhibited low levels of significance. Functional category enrichment analysis (Gene Ontology, GO and KEGG pathways) indicated a significant increase in transcription of genes responsive to Gram-positive and Gram-negative bacteria, genes of the Toll and Imd signaling pathways, and cellular heat response in Gagr^RNAi^ females (Figure 3A). In males, fewer functional categories were enriched, with immune response genes being the most prominent.

Low-expressed genes in Gagr^RNAi^ females were enriched in metabolic processes, localization, and biological processes related to interspecies interaction. In Gagr^RNAi^ males, low-expressed genes were enriched in response to biotic stimulus and lipid metabolic processes. Therefore, the most represented functional category of genes in both males and females, distinguishing Gagr^RNAi^ from control flies, was that of immune response genes, including 34 DEGs in females and 19 in males.

Under APS stress conditions, genes that increased their expression in control females showed greater enrichment in functional gene categories than in control males and Gagr^RNAi^ females (Figure 3B). Many of these categories are associated with development, including central nervous system development. This indicates that the transcriptomic response to APS is sex-specific and less pronounced in males. The knockdown of the *Gagr* gene significantly alters the response in females.

**Figure 4:**
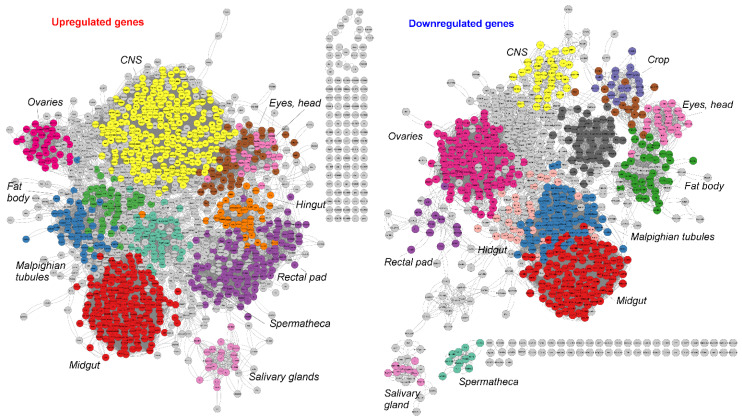
Tissue specificity of the transcriptome response to APS in the control females.

In our study of the tissue-specific response in control females, which exhibited a significant reaction to APS, we observed a systemic response encompassing numerous tissues (Figure 4). Specifically, in control females, we identified that the highest number of genes with increased expression in response to APS were predominantly active in the central nervous system and the digestive system. Conversely, the genes exhibiting the most substantial decrease in expression were those primarily active in the gut, endocrine system, and reproductive system.

#### 3.2.2. Some Genes Are Not Induced by APS Stress in Flies with the *Gagr* Gene Knockdown

**Figure 5:**
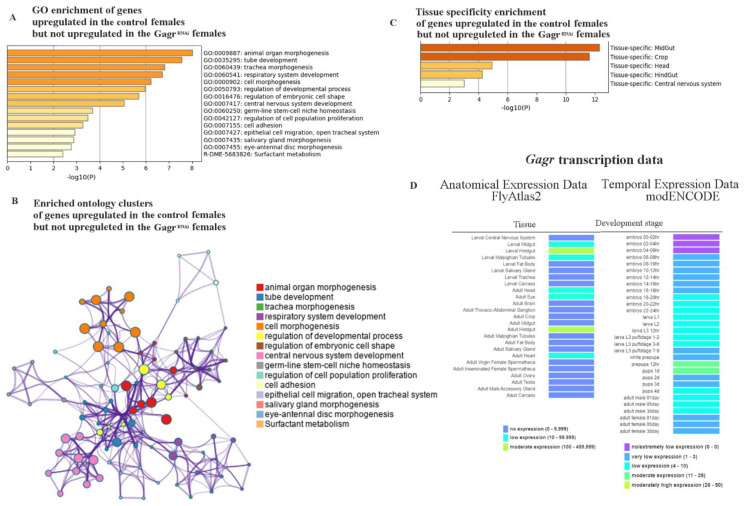
Functional enrichment of genes expressed in response to APS in the control females (LFC > 1.5, Padj < 0.05) and not activated in Gagr^RNAi^ (LFC < 0.5, Padj < 0.05). (**A**) Functional enrichment of genes (Gene Ontology). (**B**) Enriched ontology clusters (Metascape). (**C**) Tissue specificity enrichment (MCODE). (**D**) Anatomical and temporal expression data for the *Gagr* gene (FlyBase).

We next focused on identifying genes that were not activated in Gagr^RNAi^ females but showed increased expression in control females in response to stress, specifically those with a LFC greater than 1.5 for controls and less than 0.5 for Gagr^RNAi^. A total of 195 such genes were identified. Functional category enrichment analysis revealed that these genes are predominantly associated with developmental processes (Figure 5A).

Subsequently, using the MetaScape tool, we performed ontology cluster enrichment analysis for the genes overexpressed in control females (Figure 5B). This analysis involved converting a subset of representative terms into a network layout. In this network, each term is depicted as a circle node, with the node’s size indicating the number of input genes associated with that term and the color representing its cluster identity (nodes with the same color belong to the same cluster). Terms sharing a similarity score greater than 0.3 are connected by edges, where the thickness of each edge corresponds to the similarity score. This network was visualized using Cytoscape with a “force-directed” layout and edge bundling for enhanced clarity. Notably, all clusters in this network are interconnected.

The enrichment of functional categories indicated that genes activated in control females but not in Gagr^RNAi^ females show tissue specificity (Figure 5C). These genes are particularly associated with expression in the crop, midgut, hindgut, and central nervous system. Interestingly, the tissue-specific expression pattern of these genes closely mirrors that of the *Gagr* gene itself, as per the expression data available in FlyBase (Figure 5D).

In our study, we focused on identifying genes that were activated by stress in control females but remained inactive in Gagr^RNAi^ females. Functional enrichment analysis of this gene set, based on molecular function, revealed a distinct group of 19 transcription factors (Fold Enrichment: 4.76; *p*-value = 8.51 × 10^−9^, FDR = 2.53 × 10^−5^). These transcription factors include *run*, *ss*, *ase*, *sr*, *Antp*, *Sox21a*, *esg*, *grh*, *ham*, *Dfd*, *ich*, *nerfin*-1, *dmrt99B*, *grn*, *Kr*-*h1*, *acj6*, *rib*, and *tap* (Table 2). The primary biological functions of these genes’ products are associated with the development and functioning of the nervous system. Therefore, it appears that in flies with a knockdown of the *Gagr* gene, the disrupted expression of many genes may be linked to impaired activation of these transcription factors. This suggests that their activation is dependent on the *Gagr* gene.

#### 3.2.3. Transcription of Signaling Pathways Genes Is Disrupted in the Flies with the *Gagr* Gene Knockdown

Our functional enrichment analysis for genes with increased expression revealed numerous terms associated with stress response. Consequently, we specifically examined how APS influences the expression of genes involved in major stress signaling pathways.

**Figure 6:**
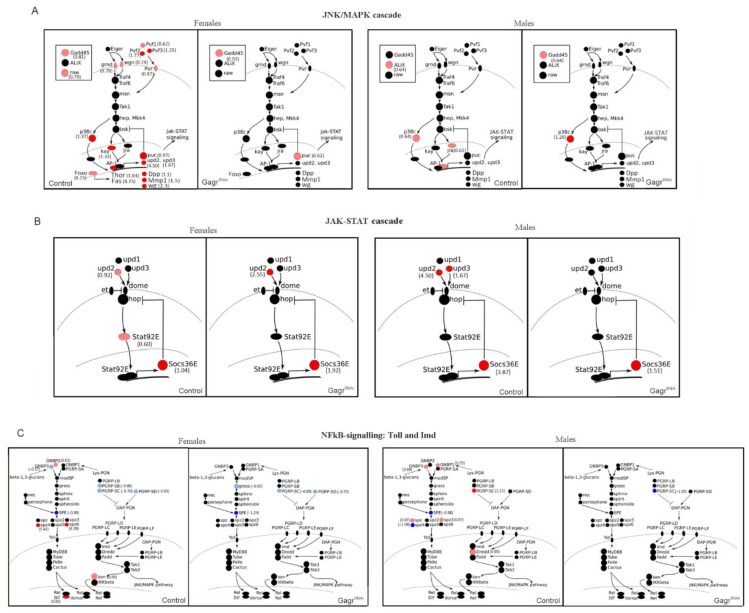
Transcription response to APS of the JNK/MAPK (**A**), JAK/STAT (**B**), Toll and Imd (**C**) signaling pathway genes in the control and Gagr^RNAi^ females and males. Genes that changed transcription by more than 2 times are shown in red (increase) and blue (decrease). Genes that changed transcription significantly, but not more than 50%, are shown in pink or light blue; genes that did not change transcription are shown in black. The LFC values are shown in brackets.

Initially, we evaluated the impact of APS on the JNK/MAPK and JAK/STAT stress cascades. In control females, APS was found to elevate the expression of key transcription factors of the JNK cascade, specifically *jra* (*Jun*) and *kay* (*Fos*) (Figure 6A). However, the key kinases of the JNK cascade did not show regulation at the transcriptional level. Other JNK cascade components that were activated at the gene expression level included *Gadd45*, which is linked to the regulation of the localization of JNK cascade proteins, and *raw*, a gene encoding a membrane protein involved in dendrite patterning and the subcellular localization of JNK signaling components. Additionally, the *puc* gene, encoding a serine/threonine protein phosphatase that forms a negative feedback loop in the JNK cascade, was also activated [22]. The genes *Pvf2* and *Pvr*, acting as a ligand and receptor, respectively, for activating the MAPK cascade, and the MAP kinase *p38c* gene, involved in stress and wound responses, also showed increased expression in response to APS [22,23]. The expression of JNK target genes *Dpp*, *Mmp1*, and *wg*, essential for cellular processes such as apoptosis and cell proliferation, was elevated as well [24]. Furthermore, the JNK pathway promotes the activity of the *Foxo* transcription factor gene, which in turn activates the expression of cytoprotective genes like *Fas1,2,3*, *GADD45*, and *Thor* [25]; these were found to be upregulated in control flies in response to APS (Figure 6A).

Therefore, it appears that the JNK cascade can be regulated at the level of expression of its components in response to APS. This regulation primarily involves the activation of the expression of extracellular ligands, their receptors, and transcription factors, but not the main kinases.

The transcriptional response to APS in Gagr^RNAi^ males and females was notably subdued. This suggests that the knockdown of *Gagr* disrupts the activation of gene expression involved in the JNK cascade.

In our analysis of the JAK/STAT signaling pathway response to APS (Figure 6B), we observed activation in the expression of certain genes in both control females and males: *upd2* (a ligand of the JAK/STAT cascade) and *Socs36E* (a negative regulator of the JAK/STAT cascade). However, the transcriptional activation of the STAT92E transcription factor was exclusively noted in control females. Therefore, the JAK/STAT cascade regulation, in response to APS, occurs at the transcription level of its components. This regulation involves the activation of cytokines and a negative regulator of the cascade. The knockdown of *Gagr* led to reduced activation of STAT92E gene expression in females and *upd2* and *upd3* in males but did not affect the *Socs36E* gene in either sex or *upd2* in females.

We also investigated the NFkB signaling pathways, Toll and Imd (Figure 6C). While their role in the innate immunity of *Drosophila* is well-documented, their protective function under abiotic stress is less understood. In control females and males, we detected no significant changes in the regulation of Imd-signaling components’ expression in response to APS, except for a modest but statistically significant increase in the transcription of the genes *ken* in females and *Dredd* in males.

For the Toll signaling pathway, however, we observed changes in the expression of several secreted factors (ligands, proteases, etc.) that positively regulate Toll signaling activity: an increase in the expression of the *GNBP2* gene, encoding the Gram-negative bacteria binding protein, and the genes *spz4* and *spz6*, involved in Toll pathway-dependent AMPs production; a decrease in the expression of the *GNBP3* gene, another Gram-negative bacteria binding protein gene, and the genes *PGRP*-*SC1a*, *PGRP*-*SC2*, and *PGRP*-*SD*, encoding peptidoglycan recognition proteins, and the *SPE* gene, coding a protease responsible for cleaving the Toll ligand. Control males also showed a decrease in the expression of several genes in response to APS.

The activation of intracellular Toll signaling components in response to APS was not detected in Gagr^RNAi^ males and females, indicating that the knockdown of *Gagr* disrupts the activation of gene expression involved in NfkB signaling pathways.

Furthermore, in control females, we noted an increased transcription level of genes activated by ER and oxidative stresses, regulated by the transcription factors Hsf1 (genes of the *Hsp70* family) and Xrp1 (genes of the *GstD* family) (Table 3). Therefore, the knockdown of *Gagr* also disrupts the activation of gene expression involved in the ER stress response.

## 4. Discussion

We observed that flies with a knockdown of the *Gagr* gene had a shorter lifespan than control flies. Additionally, the *Gagr* gene knockdown in females led to the formation of melanin capsules in the fat body. Some of the phenotypes of *Gagr* knockdown may be explained by the observed changes in gene expression. The most prominently represented functional category of genes in both sexes, differentiating Gagr^RNAi^ from control flies, is related to immune response, particularly the genes of antimicrobial peptides (AMPs).

The impact of AMP gene overexpression on the lifespan of Drosophila is a subject of mixed findings. Some studies have shown that AMP overexpression, including Drosocin and Cecropin A1, significantly extends lifespan [26], with such flies displaying reduced immune pathway activity, lesser intestinal regenerative processes, lower stress response, and delayed degradation of gut barrier integrity [27]. Conversely, other research suggests that AMP overexpression can contribute to aging through cytotoxic effects in *Drosophila* tissues [26], as chronic immune response activation may cause collateral damage to host tissues, potentially leading to premature aging and age-related diseases [28,29,30].

AMP expression is regulated by NFkB family members, including transcription factors Dif, Relish, and Dorsal [31]. Additionally, subsets of AMPs can be directly activated by the transcription factor Foxo, depending on the metabolic status of the fly, illustrating cross-regulation between metabolism and innate immunity [32]. In the midgut, AMP expression is controlled by the negative transcription regulator caudal [33,34]. However, we observed no significant changes in the gene expression of caudal and foxo transcription factors. Our data indicate activation of Toll and Imd signaling pathways in Gagr^RNAi^ flies. Consistent with the heightened activity of AMP genes, both Gagr^RNAi^ males and females exhibit shorter lifespans than control flies.

Beyond the activation of AMP genes, we observed melanotic nodules in females with the *Gagr* gene knockdown, potentially indicating autoimmune reaction induction. Of the *Ppo* prophenoloxidase family genes, only *Ppo1* showed statistically significant transcriptional changes: *Ppo1* expression is lower in females than in males for both knockdown and control flies, and its expression in Gagr^RNAi^ flies is further reduced under stress conditions. FlyBase data reveals that *Ppo1* is highly expressed in muscle cells and carcass, with overall higher expression in males than females.

Prophenoloxidase activation is partly regulated by the serine protease inhibitor Spn27A. *Spn27A* mutant larvae display melanotic phenotypes and excessive melanization in response to immune challenges [35] linked to Toll pathway activation [36,37]. Constitutive pathway activation, as in *Toll* gain-of-function or *cactus* loss-of-function mutants, leads to hemocyte overproliferation, especially lamellocytes, resulting in melanotic nodule formation [38].

Other signaling pathways can also activate melanization. Immune challenges in larvae with constitutive *PGRP*-*LE* expression upstream of the Imd pathway led to melanotic masses in the cuticle and hemolymph [39]. Activation of pathways like Ras/MAPK in hemocytes induces hemocyte proliferation and melanotic mass formation [40,41]. Constitutive JAK/STAT signaling activation, as in the dominant *Jak* mutation *hopTum-l*, induces *TotA* gene upregulation, plasmatocyte overproliferation, and lamellocyte differentiation, leading to melanotic masses in larvae and adult flies [42]. The *tuSz1* mutant shows a temperature-sensitive self-encapsulation phenotype directed at its own posterior fat body tissue [43,44], possibly due to a gain-of-function mutation in the hop gene and a loss-of-function mutation in the *GCS1* gene, disrupting the N-glycosylation pathway in the posterior fat body [45]. This demonstrates that N-glycosylated extracellular matrix proteins act as self-associated molecular patterns (SAMPs), with activated innate immune cells attacking tissues lacking these SAMPs. The self-tolerance mechanism may also initiate immunity through “missing-self recognition” if pathogens lack a self-signal on their surface [46].

Notably, *GCS1* gene transcription in Gagr^RNAi^ females and males does not change significantly, precluding a direct association of melanization with *GCS1* function in females. However, under stress conditions, *GCS1* expression significantly decreases in Gagr^RNAi^ females (LFC = −0.59, Padj = 0.0001), indicating disrupted regulation of this gene.

Our previous research has shown that transcription of the *Gagr* gene is most notably induced in females by the strong oxidant ammonium persulfate [9]. APS primarily affects membrane proteins on the cell surface, often leading to decreased cell viability and increased apoptosis [47] and causes significant oxidative stress in lysosomes, inducing epithelial-mesenchymal transition via lysosomal oxidative stress [48]. Hence, APS triggers a robust stress response, yet transcriptomic studies of APS’s effects on *D. melanogaster* are limited.

**Figure 7:**
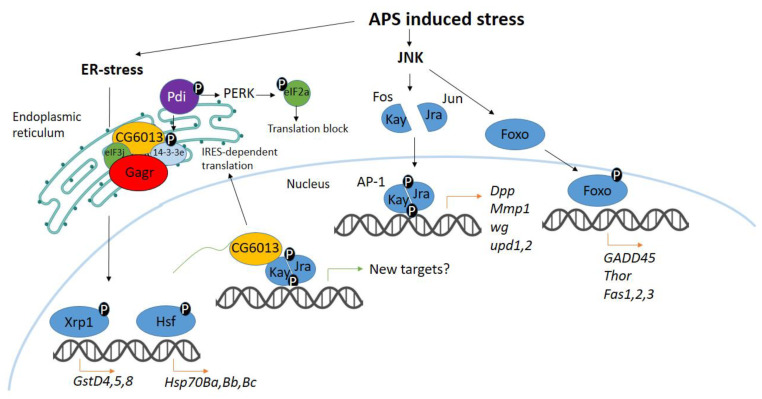
Proposed scheme for transcriptome stress response to APS. APS triggers the activation of targets within the JNK signaling pathway, including transcriptional activation of genes such as *Dpp*, *Mmp1*, *upd1*, *upd2* (which are AP-1 transcription factor targets), as well as *GADD45*, *Thor*, *Fas1*, *Fas2*, *Fas3* (targets of the Foxo transcription factor). Additionally, APS induces ER stress, activating transcription of the *GstD* family genes (targets of the Xrp1 transcription factor) and the *Hsp70B* family genes (targets of the Hsf transcription factor). However, in flies with *Gagr* gene knockdown, this transcriptional activation of the aforementioned genes is not observed.

We found that APS significantly alters the female transcriptome, activating genes associated with protective stress responses (immune response, inflammation, chitin metabolism) and suppressing genes involved in fat, protein, and carbohydrate metabolism. Stress-associated signaling pathways JNK, JAK/STAT, and Toll are regulated in response to APS (Figure 7), mainly through transcription activation.

Knockdown of the *Gagr* gene disrupts the normal activation of stress-associated signaling cascades, including JNK, JAK/STAT, and Toll. We noted that genes activated in control flies but not in Gagr^RNAi^ flies are associated with activities in the digestive and central nervous systems. Interestingly, the tissue specificity of the control response to APS correlates with the tissue-specific transcription of the *Gagr* gene.

In response to APS stress, we observed in control females an increased transcription level of genes regulated by ER stress, specifically those under the control of transcription factors Hsf1 (genes of the *Hsp70* family) and Xrp1 (genes of the *GstD* family) (Figure 7). This indicates that *Gagr* knockdown disrupts the activation of genes involved in the ER stress response.

Our findings also reveal that the transcriptomic response to stress in males is less pronounced than in females. Such sexual dimorphism in immune response is well-documented [43]. The transcription of the *Gagr* gene itself exhibits sexual dimorphism: it is about twice as high in males compared to females and is not induced by APS [9]. This lower gene activation in response to stress in males with *Gagr* knockdown could be due to a higher baseline expression of immune response genes, potentially explaining the reduced lifespan of these males under both normal and stress conditions. In control males under normal conditions, we observed increased expression of AMP genes, which might contribute to lifespan reduction, as suggested by previous studies [28,29,30].

Our data suggest that the *Gagr* gene is intricately integrated into the regulatory network of signaling cascades, with its transcription influenced by JNK and JAK/STAT pathway signals [9]. This is consistent with other studies where *Gagr* expression activation was observed in response to significant stressors like viral infection and oxidative stress. The JNK pathway plays a multifaceted role, regulating a range of processes from embryogenesis to cellular stress response. It is involved in various *Drosophila* and higher organisms processes, including apoptosis, proliferation, differentiation, cell migration, tumorigenesis, and regeneration [48,49]. The kayak protein, part of the AP-1 transcription factor, is involved in developmental regulation and may influence specific cell subsets in developing embryos [50]. In wounded tissues, JNK activation promotes apoptotic death in damaged cells and cellular reprogramming and proliferation in surviving cells [51].

JNK and JAK/STAT pathway activation in adult flies stimulate stem cell proliferation in response to oxidative or ER stress and infection [52]. JNK also regulates upd3 expression, an effector of the JAK/STAT pathway, crucial for optimal intestinal epithelium renewal and survival after septic injury [52]. In aging flies, widespread JNK activation in the intestinal epithelium induces excessive proliferation of intestinal stem cells (ISCs) [50]. Autophagy also plays a role in maintaining proliferation and preserving the ISC pool in *Drosophila*. Thus, the *Gagr* gene is observed in imago tissues with a high potential for stress-induced proliferative activity.

Transcriptomic analysis reveals that APS exposure triggers a systemic response involving multiple signaling pathways. However, in Gagr^RNAi^ flies, most signaling pathway target genes remain inactivated, indicating that *Gagr* knockdown leads to broad changes in gene expression, blocking stress response and post-stress tissue regeneration. This confirms the *Gagr* gene’s crucial role in homeostatic processes.

Considering the proven localization of the Gagr protein and its partners (CG6013, 14-3-3e, Pdi, eIF3j) associated with the ER membrane, we propose a possible scenario for the functioning of the Gagr-complex in *D. melanogaster* (Figure 7). Under oxidative or ER stress, Pdi, a redox-sensitive chaperone, first perceives the signal and transmits it to its partners [53]. The activity of Pdi and Gagr proteins may be modulated by phosphorylation, with 14-3-3e capable of binding to phosphorylated partners [54]. Concurrently, 14-3-3e can activate the Ras/MAPK pathway [55]. Gagr, possibly in partnership with 14-3-3e, binds to CG6013 and eIF3j, along with a set of mRNAs to the ribosome. The CG6013 protein, homologous to human CCDC124 and yeast Oxs1 proteins, may act as a transcription cofactor, similar to the role of Oxs1 in *S. pombe* [17].

We hypothesize that the Gagr-complex may be involved in an alternative ribosome attachment pathway to the translocon and an alternative (IRES-dependent) translation pathway under stress conditions when normal protein synthesis is hindered. This hypothesis warrants further molecular investigation.

## Figures and Tables

**Table 1:** Number of imago in crosses of the Tub-GAL4 driver with the w^1118^ and VDRC P{KK109908}VIE-260B strains.

Strain		Sum of Flies	Flies with a Short Body			Flies with a Long Body		
			Males	Females	Sum	Males	Females	Sum
tub-GAL4 × w^1118^	Observed	897	224	251	475	193	229	422
	Expected		224.25	224.25	448.5	224.25	224.25	448.5
tub-GAL4 × P{KK109908}VIE-260B	Observed	1351	315	395	710	255	386	641
	Expected		337.75	337.75	675.5	337.75	337.75	675.5

**Table 2:** Genes not activated by stress in the Gagr^RNAi^ females.

Gene	Biological Function of the Protein (According to FlyBase)
*run*	Contributes to axon guidance, dendrite morphogenesis, and germ-band extension
*ss*	Plays a key role in defining the distal regions of the antenna and the legs
*ase*	Acts together with other proneural genes in nervous system development, which involves N-mediated lateral inhibition
*sr*	Induces the fate of tendon cells in the embryo as well as in the adult fly
*Antp*	Takes a part in a developmental regulatory system that specifies segmental identity in the pro- and mesothorax
*Sox21a*	Involved in the differentiation of stem cells in the midgut
*esg*	Contributes to stem cell maintenance, tracheal morphogenesis, and neuroblast differentiation
*grh*	Responsible for the proper expression of many genes primarily involved in epithelial cell fate, barrier formation, wound healing, tube morphogenesis, and proliferation of larval neuroblasts
*ham*	Regulates neuron fate selection in the peripheral nervous system and olfactory receptor neurons
*Dfd*	Involved in proper morphological identity of the maxillary segment and the posterior half of the mandibular segment
*ich*	Regulates the transcription of factors involved in the formation of a mature apical extracellular matrix, which is essential for the integrity and shape of seamless tubes
*nerfin-1*	Regulates early axon guidance at the embryonic stage and is required for the maintenance of larval neuron differentiation
*dmrt99B*	Involved in sex differentiation
*grn*	Regulates the expression of receptors and adhesion molecules involved in axon guidance
*Kr-h1*	Involved in axon pathfinding, neurite, and axon remodeling, as well as pupal photoreceptor maturation
*acj6*	Acts in odor receptor gene expression and axon targeting of olfactory neurons
*rib*	Required for development of the salivary gland and trachea, as well as for dorsal closure
*tap*	May play a role in the specification of the sugar-sensitive adult gustatory neuron

**Table 3:** Genes of the *GstD* family and *Hsp70B* family activated by APS stress in the control but not in the Gagr^RNAi^ females.

Genes	Control Females	Gagr^RNAi^ Females
*GstD2*	LFC = 2.2, Padj = 0.01	Not activated
*GstD5*	LFC = 2.1, Padj = 0.00001	Not activated
*GstD8*	LFC = 1.6, Padj = 0.006	Not activated
*Hsp70Ba*	LFC = 3.7, Padj = 0.04	Not activated
*Hsp79Bb*	LFC = 3.4, Padj = 0.0000003	Not activated
*Hsp70Bc*	LFC = 3.3, Padj = 0.002	Not activated

## Data Availability

All data are presented and available in the manuscript. Additional information regarding the manuscript will be welcome by the authors.

## Supplementary Materials

The following supporting information can be downloaded at: https://www.mdpi.com/article/10.3390/insects15010068/s1, Table S1: RNA-seq data, Table S2: RT-PCR data, Table S3: Primers used for PCR analysis.
